# Supplementary material for: Design, Synthesis, Molecular Docking, and Tumor Resistance Reversal Activity Evaluation of Matrine Derivative with Thiophene Structure
Source: Molecules. 2021 Jan 14;26(2):417. doi: 10.3390/molecules26020417 (PMC7830115; doi:10.3390/molecules26020417)

molecules

MDPI

Supplementary information

Design, Synthesis, Molecular Docking and Tumor Resistance Re-Versal Activity Evaluation of Matrine Derivative with Thiophene Structure

Jinrui Wei [1,†], Yuehui Liang [2,†] and Lichuan Wu [3,*]

[1] Guangxi Scientific Research Center of Traditional Chinese Medicine, Guangxi University of Chinese Medicine, Nanning, Guangxi 530200, China
[2] School of Chemistry and Chemical Engineering, Guangxi University, Nanning 530004, China
[3] Medical College of Guangxi University, Nanning 530004, China
* Correspondence: wulichuan@126.com; Tel.: (+86)-771-3387126
† These authors contributed equally to this work.

Table S1. Docking score of compounds **3a-3h** and matrine against Bcl-w.

Number	Compound	London dG
**1**	**3f**	-10.9041214
**6**	**3a**	-10.629405
**4**	**3d**	-10.5346899
**7**	**3g**	-10.5202732
**3**	**3c**	-10.088953
**8**	**3h**	-10.0790882
**2**	**3b**	-9.92576027
**5**	**3e**	-9.87210655
**9**	**matrine**	-9.71206188

The HRMS, ^{13}C-NMR and ^{1}H-NMR results of 3a and 3b are reported as previously [1].

1. Li Z, Wu LC, Cai B, et al. Design, synthesis, and biological evaluation of thiomatrine derivatives as potential anticancer agents. Medicinal Chemistry Research 2018; 27:1941-1955.

The HRMS, ^{13}C-NMR and ^{1}H-NMR results of 3c~3h are displayed here.

HRMS of 3c

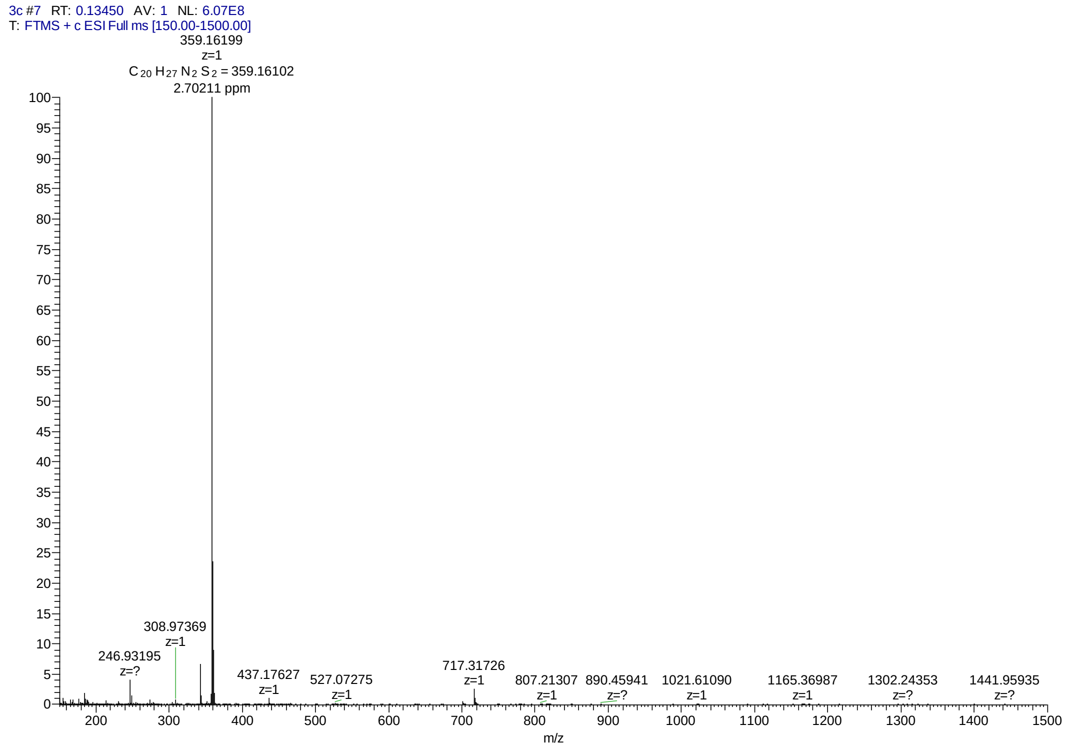

^{13}C-NMR of 3c

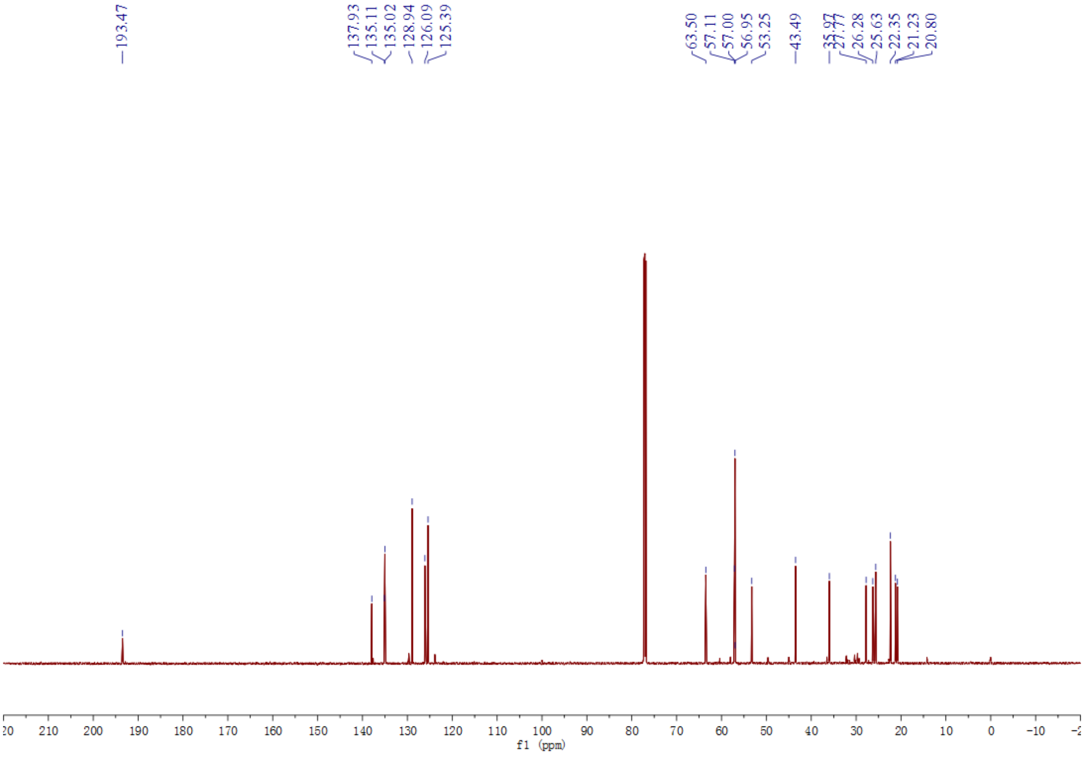

^{1}H-NMR of 3c

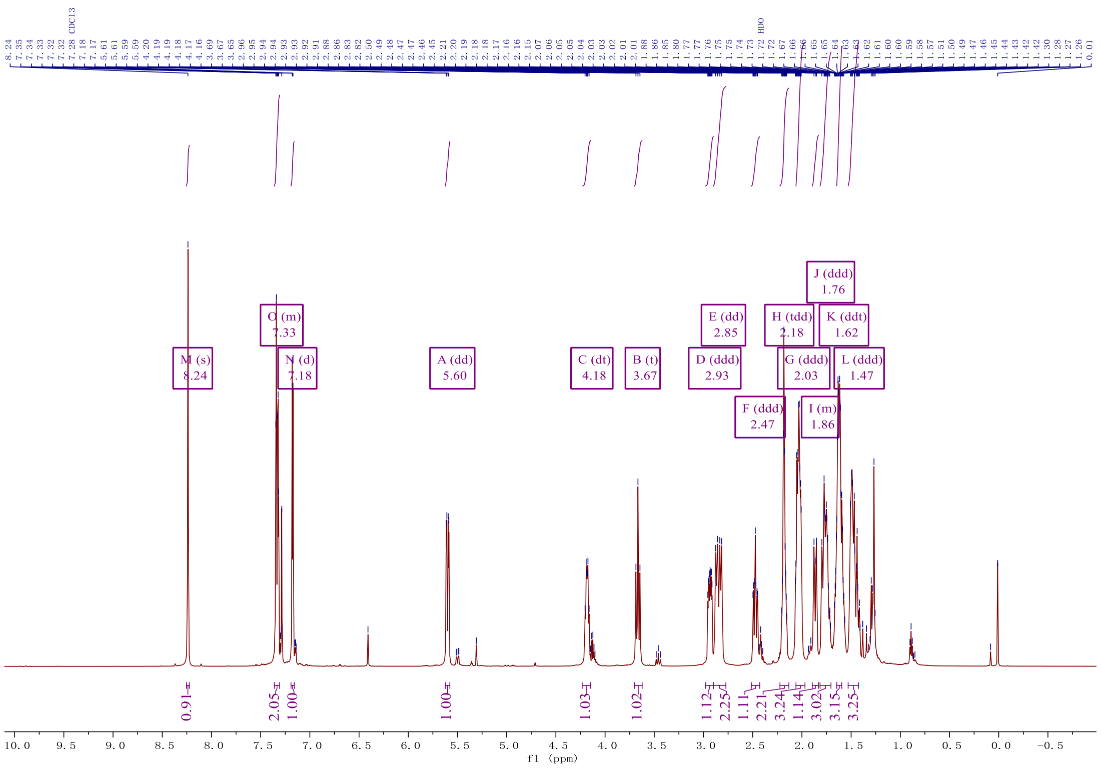

HRMS of 3d

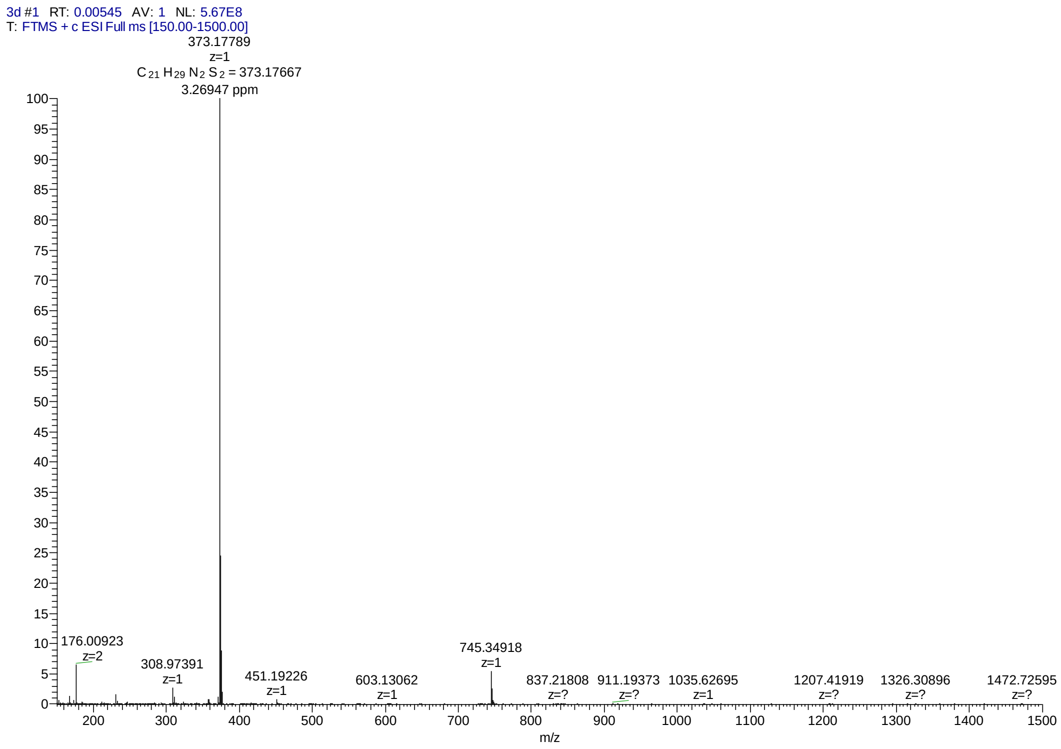

^{13}C-NMR of 3d

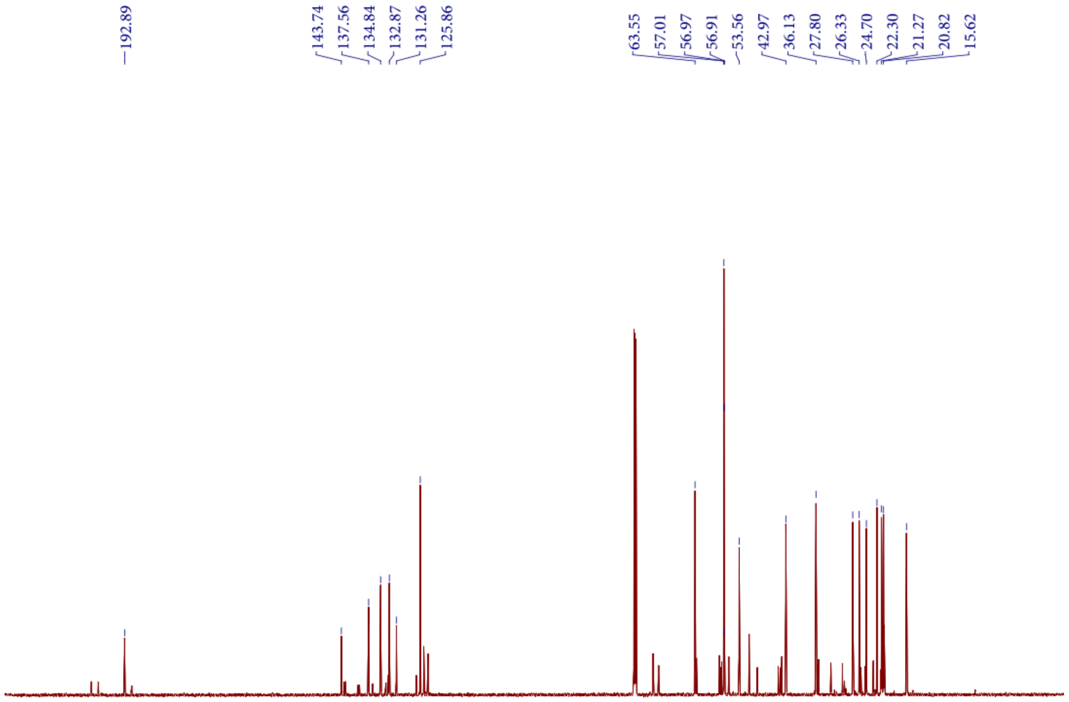

^{1}H-NMR of 3d

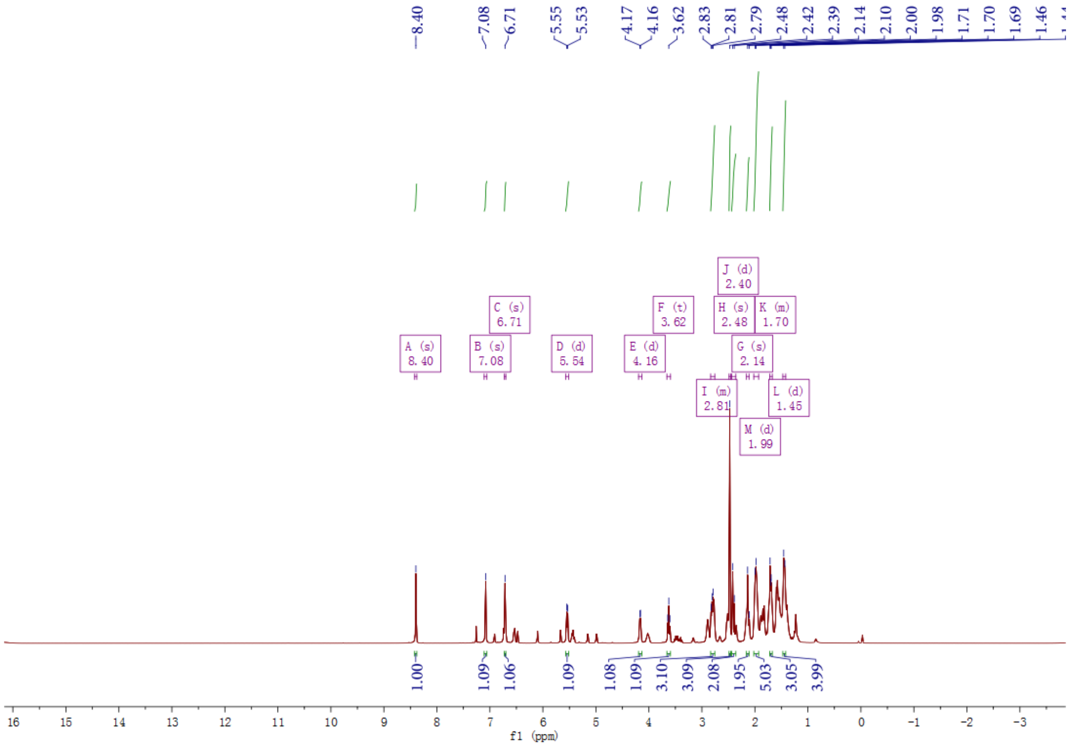

HRMS of 3e

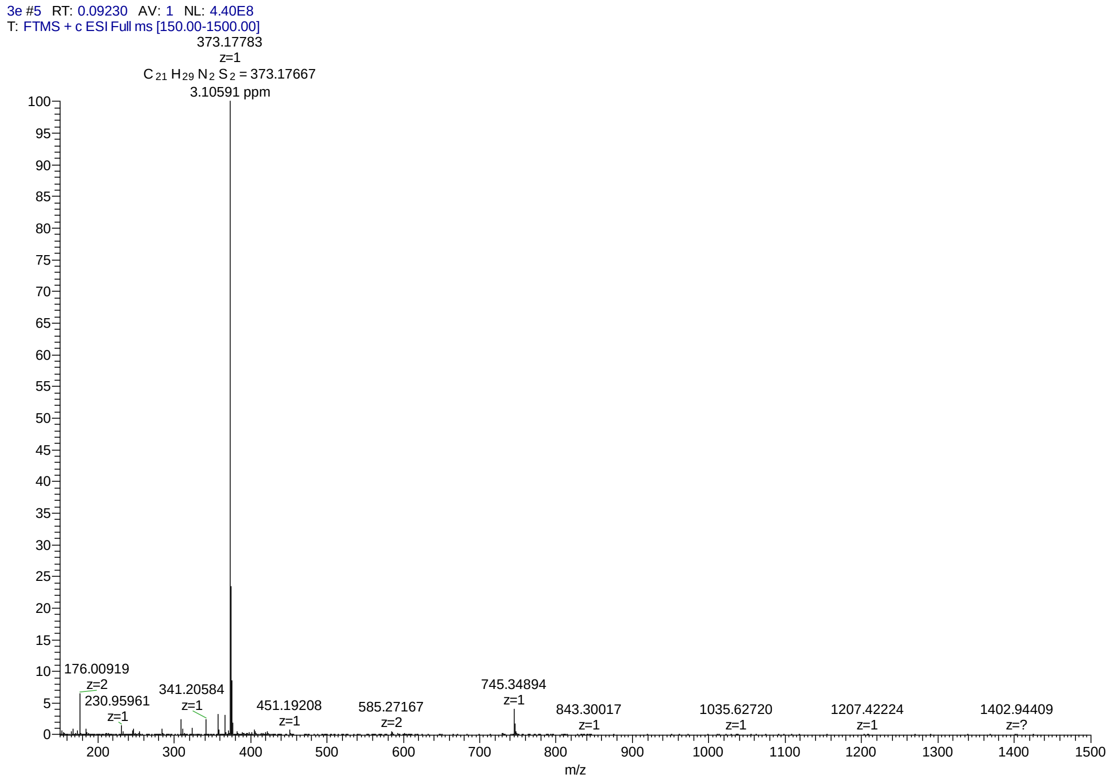

^{13}C-NMR of 3e

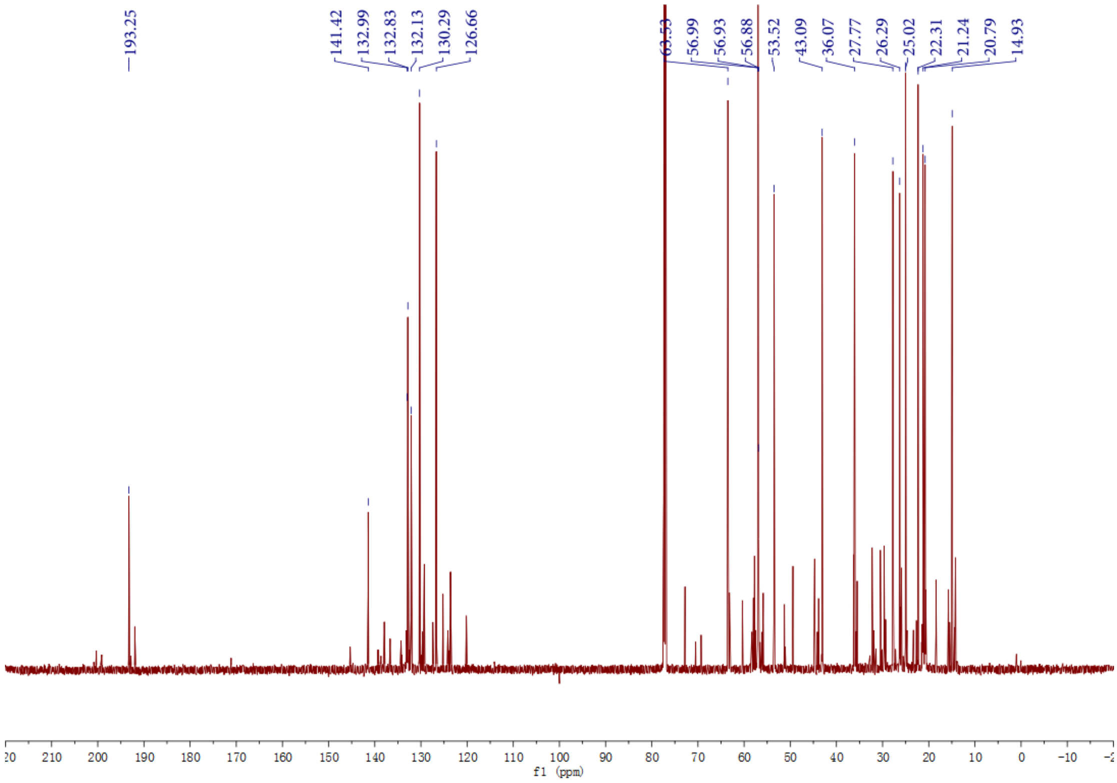

^{1}H-NMR of 3e

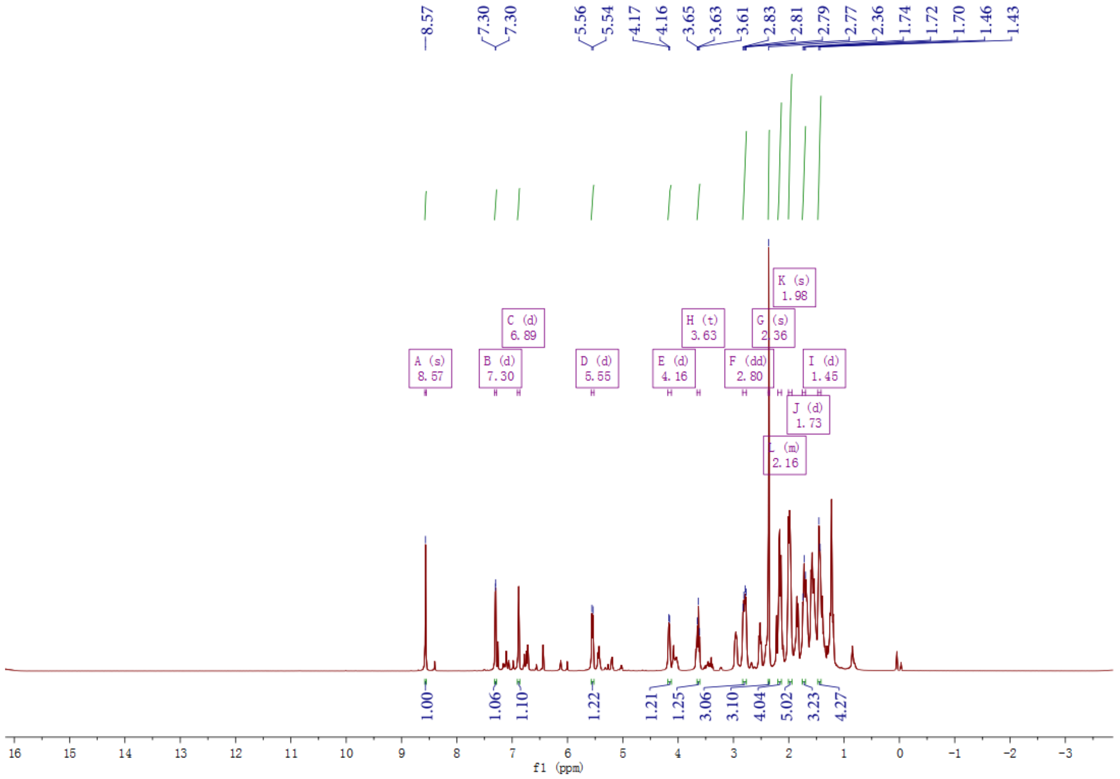

HRMS of 3f

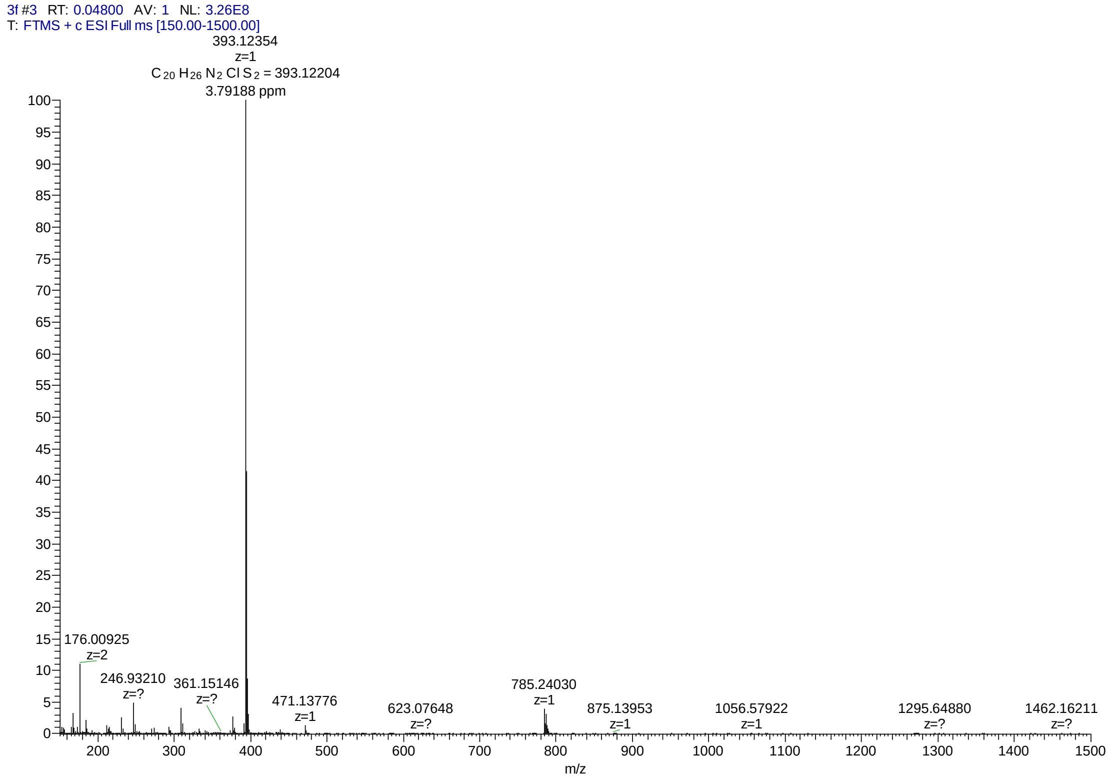

^{13}C-NMR of 3f

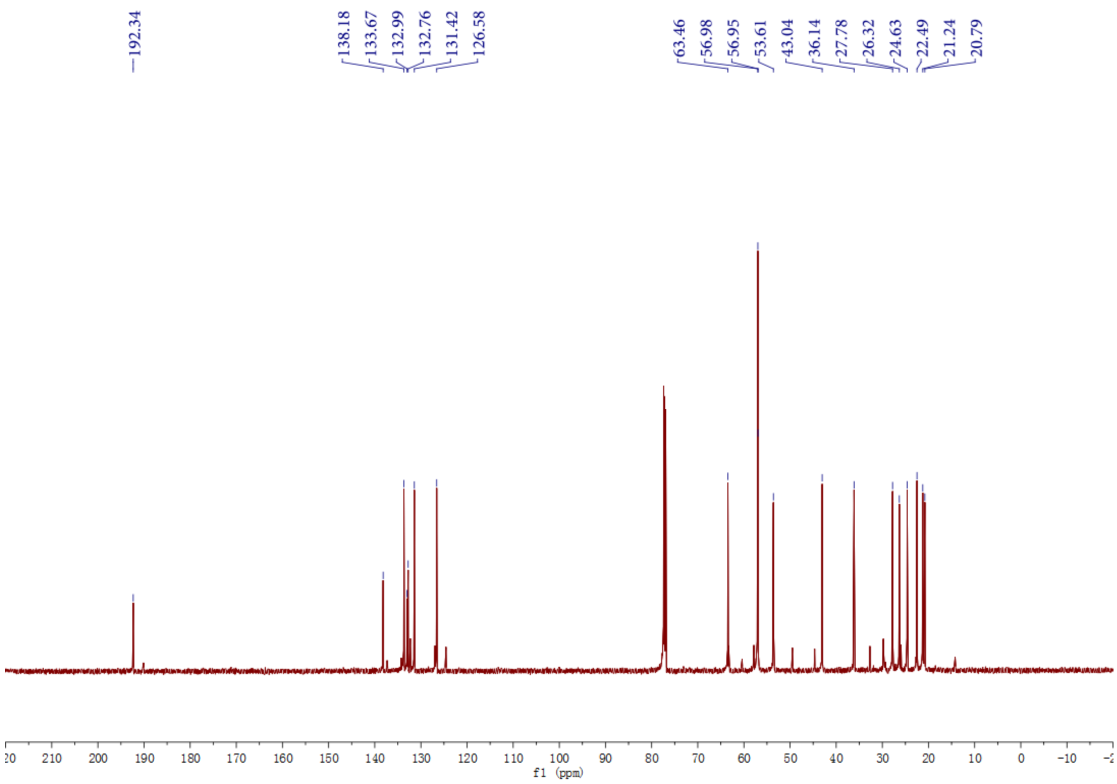

^{1}H-NMR of 3f

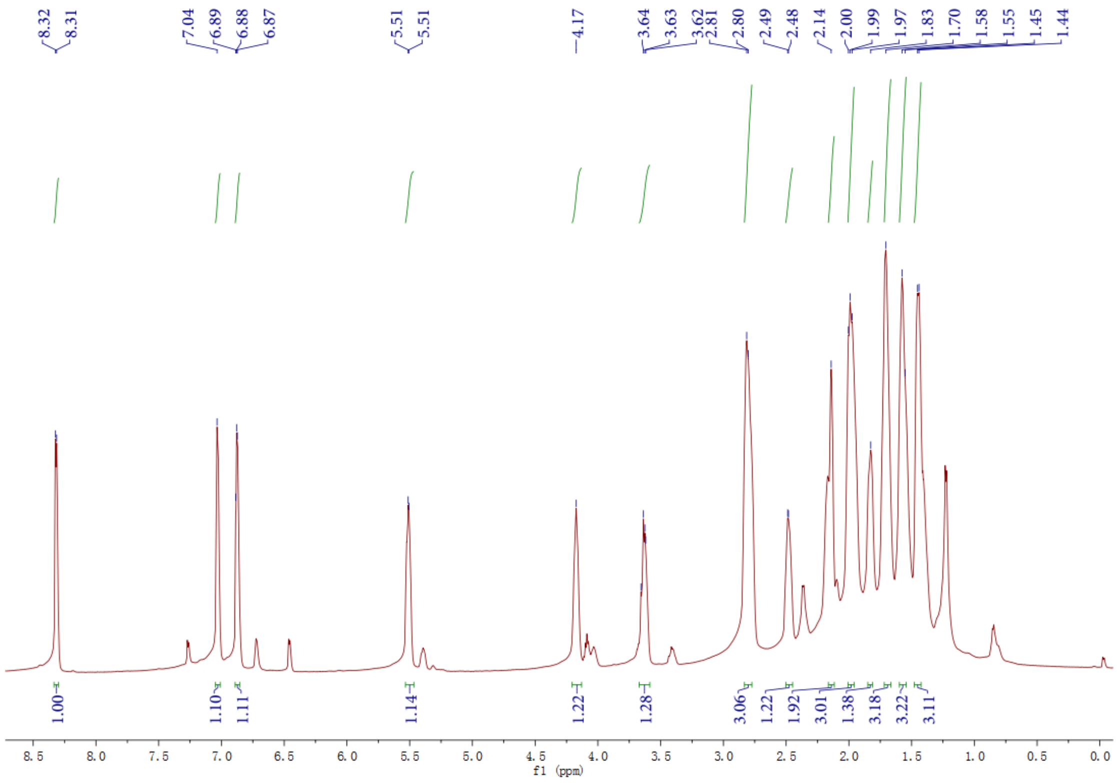

HRMS of 3g

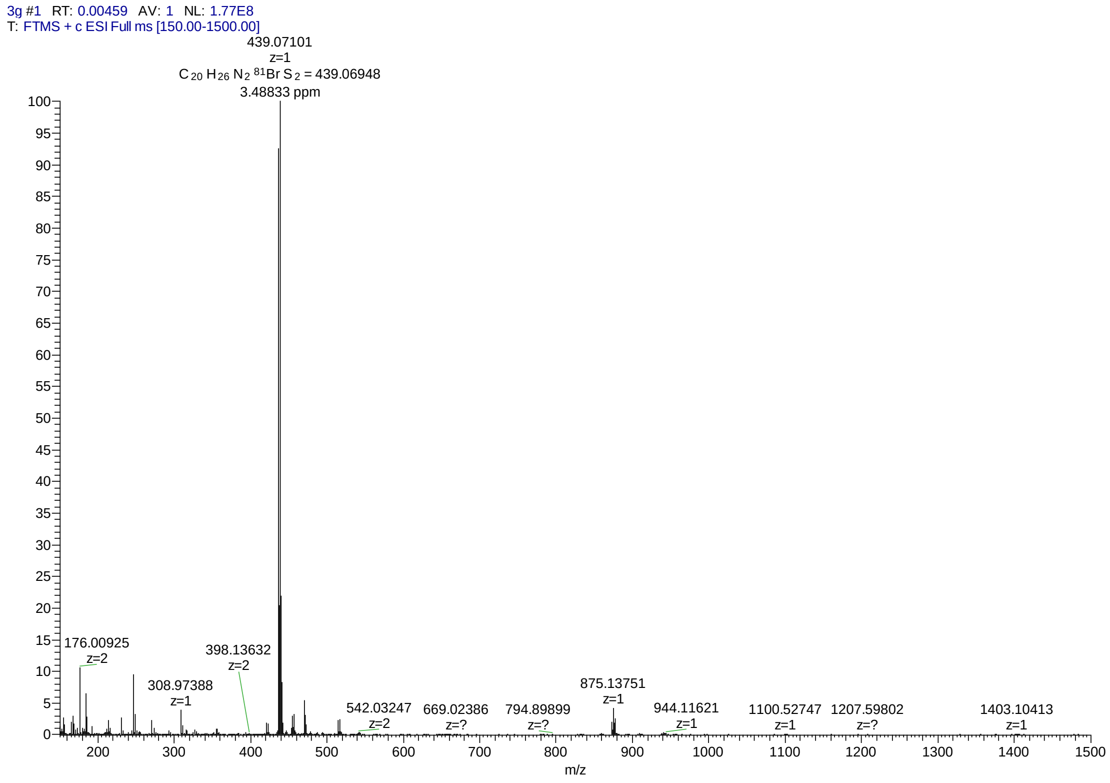

^{13}C-NMR of 3g

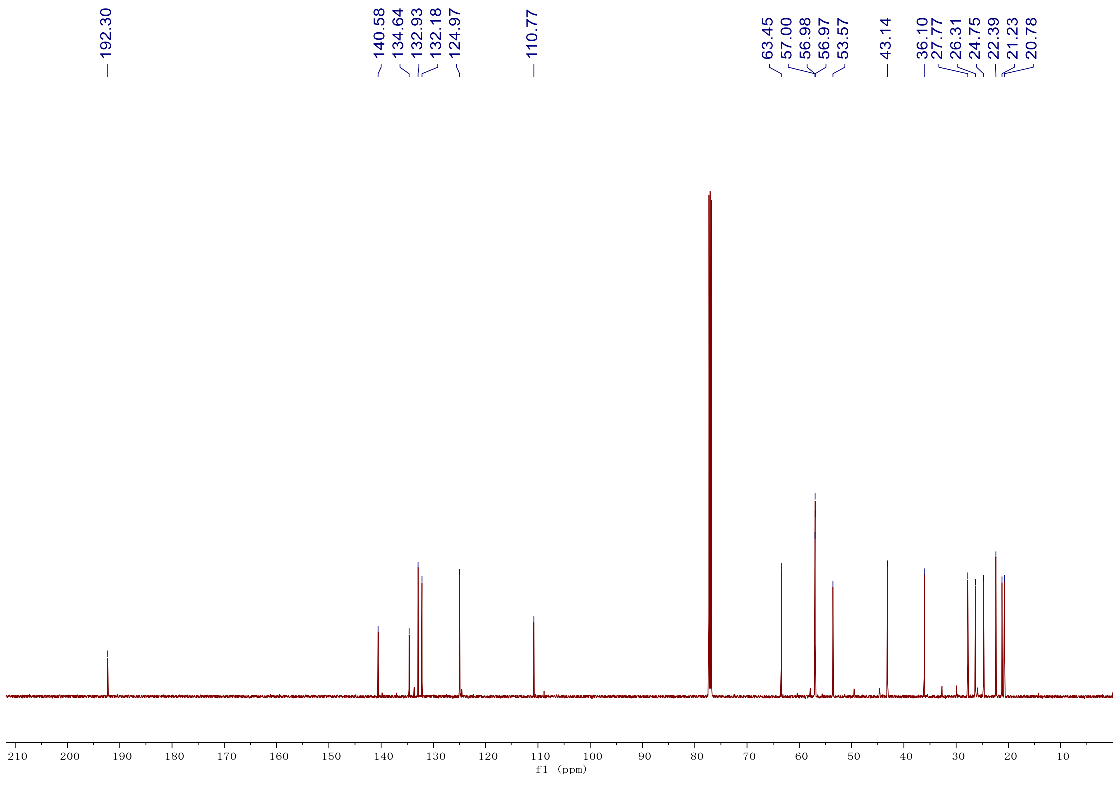

^{1}H-NMR of 3g

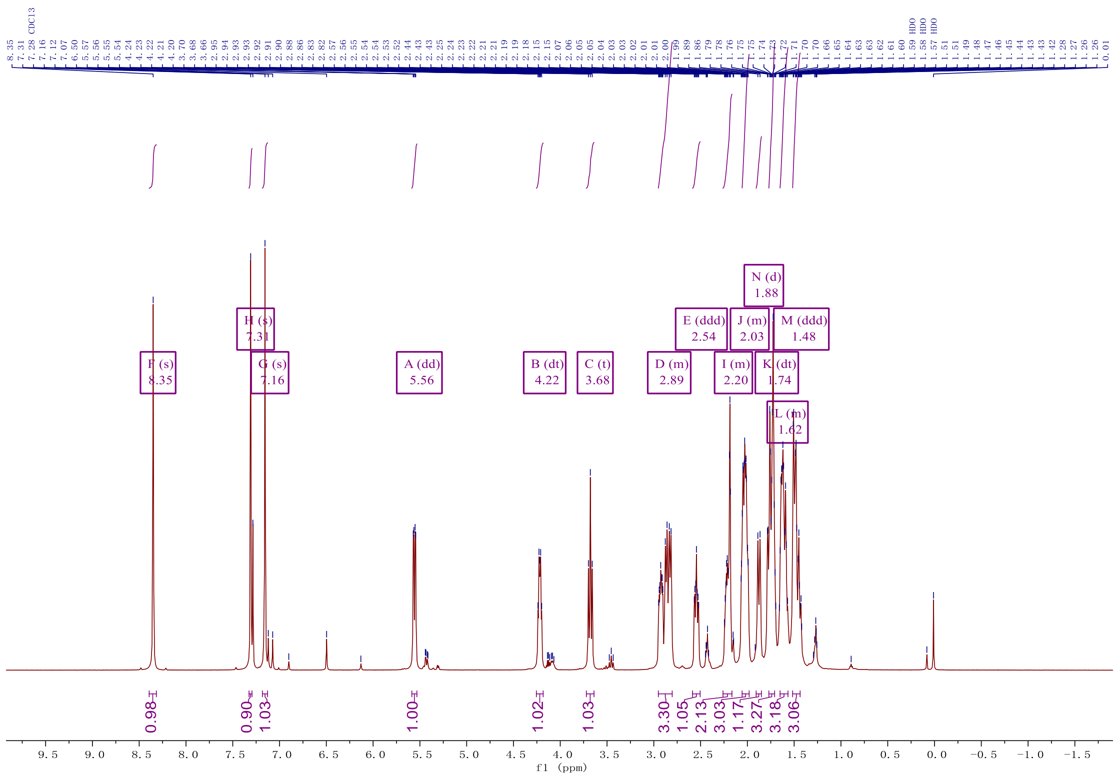

HRMS of 3h

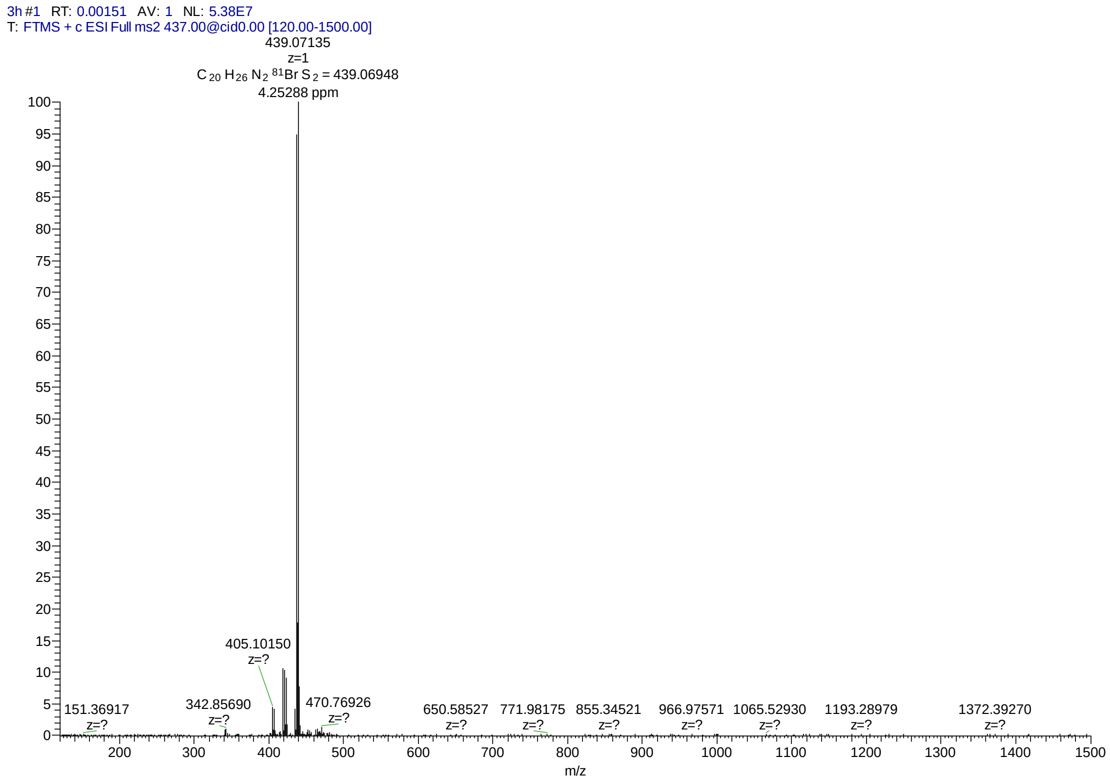

^{13}C-NMR of 3h

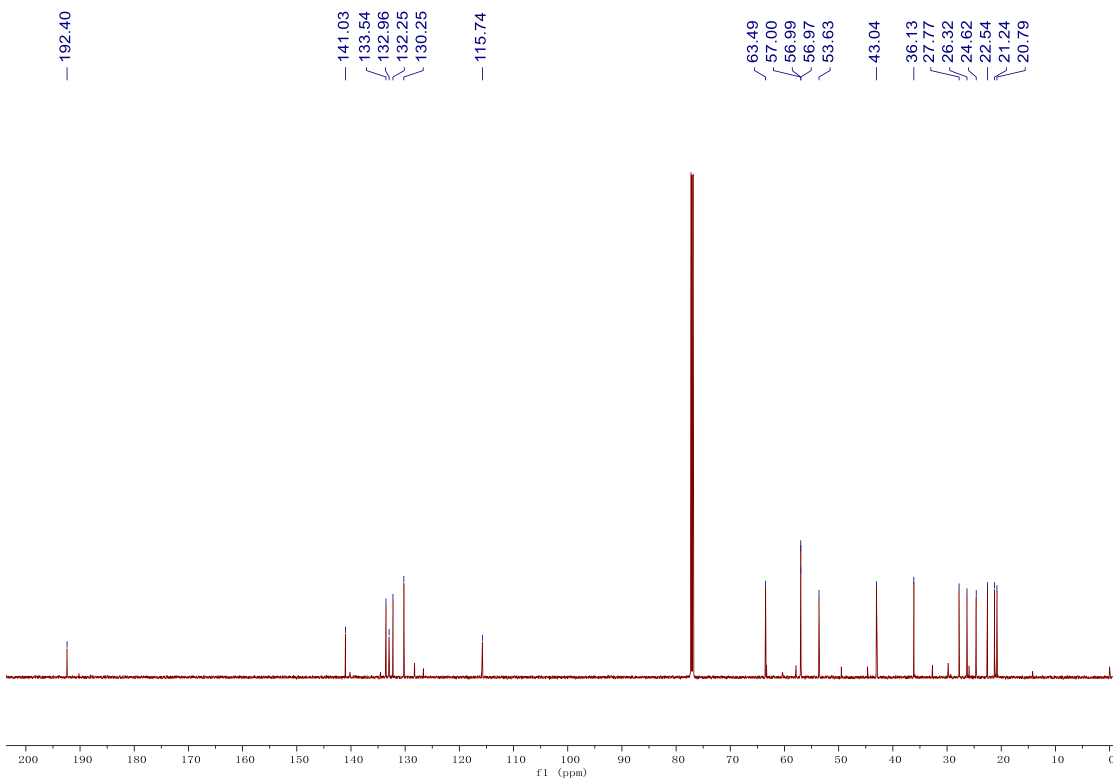

^{1}H-NMR of 3h

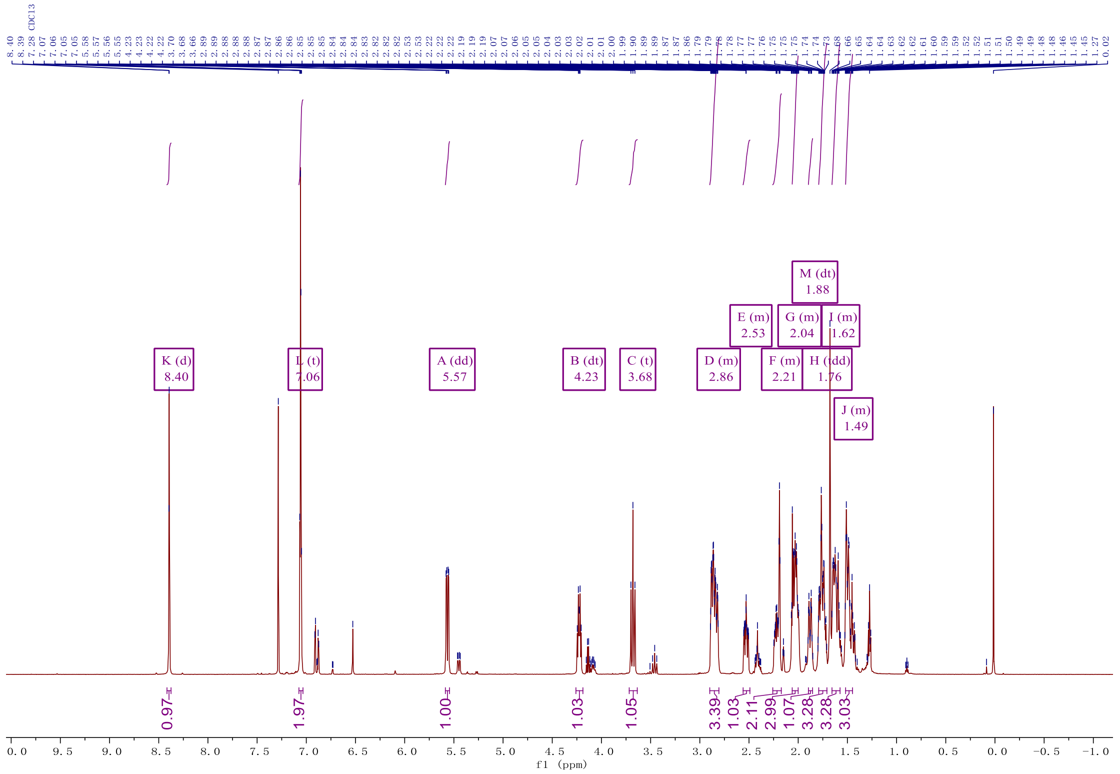

Supplement: Supplementary file 1 [file molecules-26-00417-s001.pdf]
